# Vertical Transmission of Diverse Cultivation-Recalcitrant Endophytic Bacteria Elucidated Using Watermelon Seed Embryos

**DOI:** 10.3389/fmicb.2021.635810

**Published:** 2021-11-15

**Authors:** Pious Thomas, Pramod Kumar Sahu

**Affiliations:** ^1^Thomas Biotech & Cytobacts Centre for Biosciences, Bengaluru, India; ^2^Division of Biotechnology, ICAR-Indian Institute of Horticultural Research, Bengaluru, India; ^3^ICAR-National Bureau of Agriculturally Important Microorganisms, Maunath Bhanjan, India

**Keywords:** 16S rRNA amplicon profiling, *Citrullus lanatus* (Thumb.) Matsum. and Nakai, metagenomics, seed microbial community, plant microbiome biodiversity, cultivation recalcitrant endophytic bacteria

## Abstract

Seed transmission of endophytic microorganisms is a growing research area in plant biology and microbiology. We employed cultivation versus cultivation-independent approaches on excised embryos from watermelon seeds (6–12 months in storage) and on embryo-derived *in vitro* seedlings (EIVS) to assess the vertical transmission of endophytic bacteria. Surface-disinfected watermelon seeds bore abundant residual bacteria in the testa and perisperm tissues, predominantly *Bacillus* spp. propounding the essentiality of excluding all non-embryonic tissues for vertical transmission studies. Tissue homogenates from re-disinfected seed embryos displayed no cultivable bacteria during the 1-week monitoring. Bright-field live microscopy revealed abundant bacteria in tissue homogenates and in embryo sections as intracellular motile particles. Confocal imaging on embryo sections after SYTO-9 staining and eubacterial fluorescent *in situ* hybridization (FISH) endorsed enormous bacterial colonization. Quantitative Insights Into Microbial Ecology (QIIME)-based 16S rRNA V3–V4 taxonomic profiling excluding the preponderant chloroplast and mitochondrial sequences revealed a high bacterial diversity in watermelon seed embryos mainly Firmicutes barring spore formers followed by Proteobacteria, Bacteroidetes, and Actinobacteria, and other minor phyla. Embryo-base (comprising the radicle plus plumule parts) and embryo-cotyledon parts differed in bacterial profiles with the abundance of Firmicutes in the former and Proteobacteria dominance in the latter. EIVS displayed a higher bacterial diversity over seed embryos indicating the activation from the dormant stage of more organisms in seedlings or their better amenability to DNA techniques. It also indicated embryo-to-seedling bacterial transmission, varying taxonomic abundances for seed embryos and seedlings, and differing phylogenic profiles for root, hypocotyl, and cotyledon/shoot-tip tissues. Investigations on different watermelon cultivars confirmed the embryo transmission of diverse cultivation recalcitrant endophytic bacteria. Firmicutes, Proteobacteria, Actinobacteria, and Bacteroidetes formed the core phyla across different cultivars with 80–90% similarity at genus to phylum levels. Conversely, freshly harvested seeds displayed a dominance of Proteobacteria. The findings revealed that dicot seeds such as in different watermelon cultivars come packaged with abundant and diverse vertical and seedling-transmissible cultivation recalcitrant endophytic bacteria with significant implications for plant biology.

## Introduction

Endophytic bacteria colonize plants internally without any apparent adverse effects on the host and are normally considered beneficial to the host (Hallmann et al., 1997; Afzal et al., 2019). All plants and plant parts are known to harbor bacterial endophytes with roots constituting the most widely studied region (Hardoim et al., 2015; Liu et al., 2017). Roots are also considered to be the major entry point for the organisms which traverse the cortex and reach different plant parts/organs through vascular and apoplastic channels. Other known routes of bacterial entry include the aerial plant parts such as stomata, wounds, insects, and various pollinators (Frank et al., 2017; Kandel et al., 2017). Following the realization that the majority of the soil/environmental bacteria are non-amenable to cultivation, molecular tools were applied to study the endophytic prokaryotic microbiome which revealed diverse bacterial phyla associated with the roots including several candidate phyla and some archaea (Lundberg et al., 2012; Sessitsch et al., 2012). Application of cultivation-independent molecular tools to study the endophytic bacterial diversity in the shoot tissues and in particular to the *in vitro* plant cultures which could be guarded against the external organisms revealed a huge bacterial diversity similar to the root system prevailing a cultivation recalcitrant form (Thomas and Sekhar, 2017; Thomas et al., 2017). This raised a question about the prospects of endophyte entry over and above the generally described routes.

Of late, considerable information is emerging on seed-associated microorganisms and the possibility of vertical transmission of bacterial endophytes (Nelson, 2018; Shahzad et al., 2018; Verma et al., 2019; White et al., 2019; Abdelfattah et al., 2021). Seed-associated bacteria could be of different types: (i) external on the seed surface, (ii) internal to the seed coat, or (iii) inside the embryo (Frank et al., 2017; Nelson, 2018). Seed-inhabiting bacteria possess the advantages of quick colonization at germination with some organisms possibly turning endophytic colonizers in seedlings (Cope-Selby et al., 2017; Verma et al., 2019). Studies investigating seed endophytes often used direct seeds (Barret et al., 2015), seed wash solutions (Torres-Cortés et al., 2018), mere surface-washed seeds (Adam et al., 2018), or in most instances surface-sterilized whole seeds (Khalaf and Raizada, 2016; Bergna et al., 2018; López et al., 2018). While most of the past studies adopted the cultivation-based approach (Truyens et al., 2015), some employed cultivation-independent molecular tools (Cope-Selby et al., 2017; Chen et al., 2020) with or without assessing the effectiveness of surface sterilization. In cultivation-based studies, generally Proteobacteria formed the dominant phylum with *Bacillus* spp., and other spore-forming Firmicutes also making a dominant constituent (Truyens et al., 2015; Khalaf and Raizada, 2016; Nelson, 2018).

Most studies on seed-associated bacteria did not make a discrimination between embryo inhabitants and those in other seed parts. Only the embryo-colonizing endophytes perhaps bear the advantage of vertical transmission between successive plant generations, while several bacteria could get incorporated from the seed spermosphere at germination (White et al., 2014; Shade et al., 2017; Shahzad et al., 2018). Additionally, the embryo colonizers are certain to reach different parts of new seedlings and adult plants. It is essential to distinguish the embryo-associated microbiome from other seed microbiota to elucidate the vertically transmissible microorganisms. Very few studies have factually examined the plant microbiome biodiversity confined to the embryos *per se*. A cultivation-based study on seed-endophytes in tomato employing *in vitro* grown seedlings where the seed coat could be separated post-seed germination displayed some embryo-associated bacteria that apparently arose from the activation of cultivation recalcitrant endophytic bacteria (CREB) constituting mainly Proteobacteria (Shaik and Thomas, 2019). Molecular analysis on surface-sterilized tomato seeds showed a highly diverse bacterial biome, but it was not possible to conclude whether the organisms were embryo colonizers or inhabiting other seed tissues (Thomas and Shaik, 2020). Investigations on wheat endosperm versus excised embryos revealed evident embryo colonization by a few bacterial genera (Kuźniar et al., 2020a, b). A recent internal transcribed spacer (ITS) region and 16S rRNA gene amplicon-based study of fungal and bacterial community separately in the embryo and pericarp of oak acorns showed twofold to fourfold higher microbial diversity and richness in the embryo than in the pericarp with some 20 phyla of the bacterial community in the embryos that included transient and seedling-transmitted microbiome (Abdelfattah et al., 2021).

Microscopy forms an essential tool to establish the presence of bacterial endophytes inside the tissues; this information is often lacking in the studies on endophytic microbiome. Microscopic explorations on banana and papaya shoot tissues and *in vitro* cultures indicated abundant intracellular bacteria with no obvious bacterial presence in the inter-cellular regions, which appeared contrary to the general perception about the bacterial endophytes as inter-cellular colonizers (Thomas and Sekhar, 2014; Thomas et al., 2019). The term “Cytobacts” was coined to describe such cultivation-recalcitrant cytoplasmic colonizers which were also demonstrated in long-term actively maintained cell and callus cultures of different plant species comprising a huge taxonomic diversity as documented with fluorescent *in situ* hybridization (FISH) and 16S rRNA amplicon profiling on grape cell cultures (Thomas and Franco, 2021). The intracellular bacterial associations and the huge taxonomic diversity documented across plant species strengthened the possibility of vertical transmission of bacteria from one generation to the next.

Watermelon seeds bear the advantages of the embryo being well protected inside the hard testa and the feasibility of embryo excision excluding all external tissues. Watermelon seeds are generally dried before long-term storage bringing the moisture content down to about 7–8%. The extreme desiccation and high osmotic potential during drying and subsequent storage (ambient or low temperature) make the bacterial survival extremely difficult. Only spore-forming bacteria are generally considered to withstand such extreme conditions (Nelson, 2018). Studies addressing the seed microbiome in cucurbits (Khalaf and Raizada, 2016; Adam et al., 2018; Glassner et al., 2018) did not make the effort to separate the embryos from other seed parts. The extent of organisms that survive the desiccation and transmit to the next generation is best studied by analyzing the embryo-derived seedlings under sterile conditions. Tissue culture systems offer the feasibility of growing the embryo-derived seedlings *in vitro* protected from external organisms and also help in monitoring the distribution of true-embryo-associated bacteria in developing seedlings. This study was aimed at assessing the seed and embryo association of endophytic bacteria in stored dry seeds employing watermelon seeds and *in vitro* grown seedlings as the experimental system.

## Materials and Methods

### Seed Material and Experimental Approach

Seeds of watermelon cv. “Arka Manik” (ICAR-Indian Institute of Horticultural Research, Bengaluru) were employed in detailed experiments, while other watermelon cultivars were used in extended studies. In general, “Arka” series seeds were procured from the institute seed sales counter packaged in standard polymer seed bags and stored under ambient conditions or at 16°C. After procurement, seeds were used immediately or were refrigeration stored (4°C). The seeds as per the date of packaging were of 1–6 months old, and were used within 6–12 months as mentioned under specific experiments. The seed lots were periodically checked for viability recording minimum 90% germination.

The study involved assessing the surface sterilization needs to ensure the aseptic excision of embryos, microscopic elucidation of embryo-colonization, and cultivation versus 16S rRNA metagene V3–V4 phylogenetic analysis on seed embryos and axenically grown seedlings. This was followed by V3–V4 taxonomic profiling on different parts of embryos and on seed embryos of four watermelon cultivars. All experiments were carried out under aseptic conditions with strict measures to avoid the lateral entry of microorganisms (Thomas and Sekhar, 2017). Unless mentioned differently, nutrient agar (NA) prepared in single-use γ-irradiated 90-mm plates and monitored for 4–5 days for sterility assurance after sealing in polypropylene (PP) bags was used for cultivation-based bacterial monitoring. For tissue homogenate preparation and serial dilutions, sterile 0.2-μm filtered distilled water post-autoclaving (FDW) was employed with sterility confirmation at use. The baseline to define the endophytic microbiome as CREB constituted the absence of bacterial colony growths from the tissue homogenates applied on NA or trypticase soy agar (TSA) at different serial dilutions with 36–37°C incubation for one night to trigger the growth and thereafter for 1 week under ambient conditions (at 26–30°C to reduce the chances of fast-spreading colonies) when the corresponding sample displayed high bacterial diversity/abundance as per deep sequencing.

### Cultivation-Based Assessment of Seed-Associated Bacteria and Surface Sterilization Needs

An initial assessment of the extent of bacterial association with dry seeds and the surface sterilization needs was undertaken through different disinfection steps and monitoring the seed wash solutions pre- and post-chemical treatments using 6-month refrigeration-stored watermelon “Arka Manik” seeds. Precisely, 100 seeds were vortexed for 20 min in 10 ml FDW (0.01% Tween-20) in a 50-ml Falcon tube, and the colony-forming unit (CFU) released was assessed through single plate-serial dilution spotting (SP-SDS) (Thomas et al., 2015) of wash solutions on NA employing four replications. Seeds were further rinsed five times in FDW (100 μl per seed) with the wash solution monitoring through 10 μl sample spotting on NA. After this step, 25 seeds each were taken through three treatments: (T1): six washings using FDW with the first step in 0.01% Tween-20, then 1 min treatment with 90% ethanol; (T2): T1 followed by chemical disinfection for 5 min employing NaOCl (4% available chlorine; Fischer Scientific); or (T3): T1 succeeded by 0.1% HgCl_2_ (0.1% Tween-20) treatment, with wash solution monitoring by spotting as above. After six rinses in FDW, seeds were aseptically dried on tissue paper and imprinted on NA. The dried seeds were decoated aseptically using a sterile nail cutter, and the seed coat tissues were assessed for any cultivable bacteria after an extended manual homogenization (15–20 min) in a mortar (50 mg ml^–1^ FDW) followed by SP-SDS. The excised seed embryos (25 each) with the adhering perisperm membrane were monitored for external bacteria after 20 min gentle vortexing in FDW (0.01% Tween-20) followed by six rinses (2.5 ml each time). Finally, the seed embryos were homogenized in a mortar (100 mg ml^–1^ FDW), and the bacterial CFU was assessed through spotting-and-tilt-spreading (SATS) (Thomas et al., 2012) on NA, which allowed 100 μl samples, and through SP-SDS on TSA, a cost-saving reliable method. The plates were monitored for 1 week at 36–37°C for one night followed by 30°C, as above. As per the outcome, the disinfection method employing NaOCl (4% chlorine) was tested again with 1- and 12-month refrigeration-stored seed lots. Surface-sterilized embryos were cultured on sugar-free Murashige and Skoog (MS) medium (Murashige and Skoog, 1962) gelled with 0.1% Phytagel (Sigma Chemical Company, St. Louis, MO, United States) to assess the effect of the chemical treatments on seed germination. Based on the results, excision of seed embryos followed by their surface sterilization with perisperm exclusion as per T2 above was adopted to study the embryo-associated microbiome.

### Microscopic Observations on Seed Wash Solutions, Tissue Homogenates, and Tissue Sections

Seed soak/wash solutions from surface-sterilized watermelon seeds, seed testa homogenate, perisperm membrane, and the embryo sections were examined under bright field/phase contrast using a Leica DM2000 microscope, and still images/movie were captured under high magnification (×1,000) as described elsewhere (Thomas and Sekhar, 2014). Further, thin sections from dry seed embryos were prepared with a cryostat microtome (Thermo Scientific Microtome FSE) or with a razor blade after 1–2 h soaking in FDW and were examined in a Leica LB-02 epi-fluorescence microscope after staining with SYTO-9 and propidium iodide employing the Live/Dead bacterial staining kit (Molecular Probes) as per Thomas and Sekhar (2014). Razor-thin sections prepared from FDW-soaked embryos were further examined under confocal scanning laser microscope (Nikon Confocal A1, 90i) after staining with SYTO-9 with the imaging done under 488 nm green channel.

Fluorescent *in situ* hybridization was undertaken on fixed tissue of watermelon seeds (4% formaldehyde solution) as per Rossmann et al. (2012), adopting the basic protocol of Pernthaler et al. (2001) using 5′ Cy3 labeled Eub338 probe and Eub338ns control probe. The imaging was done under 543.5 nm channel using the NIS element 3.2.3 program (Nikon). Eub338 FISH images were captured under the same settings where no signal was observed with the Eub338ns probe.

### Cultivation Versus 16S rRNA Amplicon Profiling of Seed Embryo/Seedling-Associated Bacteria

Embryos excised from dry seeds were disinfected with NaOCl (4% chlorine) as per the optimized procedure with CFU monitoring of all wash solutions. The embryos were cleared off the perisperm tissue, treated with filter-sterilized 2% Na_2_S_2_O_3_ (10 min) to remove chloramines, washed twice in FDW, and imprinted on NA to ensure proper surface sterilization. After aseptic weighing, 50 embryos were homogenized in a mortar (1 ml FDW/embryo; approximately 40–50 mg per embryo), and the decimal dilutions (10^0^ to 10^5^) of the homogenate were applied on NA as per SATS and on TSA through SP-SDS. The plates were incubated at 37°C (NA) or 30°C (TSA) to provide wider growing conditions.

#### Watermelon Seed Embryos Versus *in vitro* Seedlings

This study involved DNA extracted from excised and surface-sterilized seed embryo (MG-37) and 2-week-old seed embryo-derived seedlings (MG-41) of watermelon cv. Arka Manik. The *in vitro* seedlings were confirmed to be free from cultivable bacteria through their indexing on NA and TSA (Thomas and Sekhar, 2017). DNA was extracted from the milky seed embryo homogenate or the *in vitro* grown index-negative seedlings (after testing for any cultivable bacteria) employing PowerFood (PF) microbial DNA isolation kit (MOBIO Laboratories, Inc., Carlsbad, CA, United States). After preliminary quantity and quality assessments, the DNA was submitted to M/s Xcelris Labs Ltd., Ahmedabad^1^ for 16S rRNA gene taxonomic profiling. 16S rRNA gene amplicon libraries were prepared by M/s Xcelris targeting the V3–V4 hypervariable region as per the standard Illumina 16S Metagenomic Sequencing Library preparation protocol. Library preparation, PCR amplification, amplicon purification, paired-end sequencing on Illumina MiSeq platform (2 × 300 bp), quality filtrations, chimera screening, stitching, and the operational taxonomic units (OTU) picking were undertaken as described by Thomas and Sekhar (2017) with bioinformatics support from the service provider. For taxonomic assignment, QIIME bioinformatics analysis tool was employed based on sequence similarity within the reads in Greengenes database through *de novo* approach excluding singletons (<2 reads). Two rounds of QIIME analyses were undertaken to avoid the majority of reads corresponding to chloroplast and mitochondrial 16S rRNA with the second round analysis (QIIME analysis II) excluding the plant sequences and unassigned reads from the sequence files as described in detail elsewhere (Thomas and Sekhar, 2017).

#### Whole Embryos Versus Embryo-Base and Shoot Tip + Cotyledon Parts of Embryos

In this study, 20 re-disinfected whole seed embryos were employed in comparison with another 20 embryos where the embryo-base (comprising the radicle and plumule which is to develop as the new seedling) and cotyledon parts were segregated and used for DNA isolation. The DNA samples after the quality and quantity assessments were taken through 16S rRNA gene taxonomic profiling as above.

#### 16S V3–V4 Taxonomic Profiling of Watermelon Seedling Root, Shoot, and Hypocotyl Parts

The root, hypocotyl, and the remaining shoot tissues comprising the shoot tip and the cotyledons from 2-week-old *in vitro* grown seedlings post-surface sterilization and after assessing the effectiveness of surface sterilization were employed here. The tissue homogenates after serial dilutions were plated separately on NA to assess the cultivable bacterial population. DNA was extracted from the three tissue homogenates separately using the PF kit, and the samples were submitted to M/s Xcelris Labs (after the quality and quantity assessments) for the 16S V3–V4 taxonomic profiling.

#### 16S V3–V4 Taxonomic Profiling on Seed Embryos of Four Different Watermelon Cultivars

Seeds of four watermelon cultivars, “Arka Manik,” “Arka Muthu” (ICAR-IIHR), “Madhubala” F1 Hybrid, and “SS455” (Nunhems, Bangalore) stored at 4°C were employed here. The former two had small black seeds, while the latter two showed large bold seeds. Seed embryos were gathered after seed decoating and surface sterilization and were cleared off the perisperm membrane. The embryo homogenates were tested for cultivable bacteria on NA and TSA through SP-SDS. The embryo homogenate suspension was stored at 4°C for 1–2 h to allow the large particles to settle down. The supernatant was subjected to three rounds of spinning, and the DNA was extracted from the pellet employing the PF kit. The 16S rRNA V3–V4 taxonomic profiling was performed as above.

### Experiment Setup and Statistical Procedures

For surface sterilization trials, 100 seeds of different lots were employed. For cultivation versus cultivation-independent assessment of embryo microbiome, the homogenate derived from about 50 excised seed embryos (homogenized in 1 ml FDW per seed embryo) was used. Cultivation-based studies were targeted at getting an estimate of the cultivable bacteria which employed four replications for different serial dilutions. Deep-sequencing studies were undertaken on DNA samples pooled from different seed lots with a single replication per sample. The deep-sequencing data generated in this study have been deposited with the National Center for Biotechnology Information/Sequence Read Archive (NCBI/SRA).

### Accession Numbers

The metagenome data generated have been deposited with NCBI/SRA under the project title “Cucurbit Seed, Embryo and Seedling Microbiome” with the bioproject ID: PRJNA564696, BioSample accession nos. SAMN12726399 to SAMN12726402 (MG37-MG40); SAMN12726475 to SAMN12726478 (MG41-44) and SAMN12726555 to SAMN12726558 (MG45-48) as indicated in the respective tables.

## Results

### Seed Bacterial Load and Surface Sterilization Needs

Preliminary observations on watermelon “Arka Manik” seeds surface-sterilized with NaOCl showed residual bacteria in the seed coat as per the monitoring of wash solutions. Surface sterilization involving ethanol soaking and NaOCl treatment also did not eliminate all the external bacteria. Monitoring the seed external and internal bacterial load after the three treatments, namely, repeated FDW washing followed by 1 min ethanol soaking (T1), T1 followed by 5 min NaOCl treatment (T2), and T1 succeeded by 5 min 0.1% HgCl_2_ (T3), indicated a large amount of seed external bacteria (1.0 × 10^4^ CFU/seed), a part of which (4.4 × 10^3^ CFU/seed) was released with mere water-rinsing which comprised largely spore-forming *Bacillus* spp. (Table 1 and Supplementary Figure 1).

**TABLE 1:** Bacterial CFU detected with 6-month refrigeration-stored seeds of watermelon “Arka Manik” adopting different surface disinfection methods.

Step	Particulars			CFU per seed
A	Direct seed washing: 100 seeds from a 6-month-old seed lot were vortexed for 20 min in 10 ml FDW (0.01% Tween-20) and the solution monitored for bacterial CFU			4,400

B	Seed washes 2–6: Seeds were further rinsed five times in 10 ml FDW (100 μl per seed)			

	Rinse 1			517
	Rinse 2			296
	Rinse 3			282
	Rinse 4			197
	Rinse 5			93

	Total CFU removed/seed (A + B)			5,685

C	Surface-disinfection treatment followed by the monitoring of six wash solutions	T1: Mere 90% ethanol soaking for 60 s	T2: Ethanol (90%) treatment (60 s) followed by 5 min NaOCl treatment (4% available chlorine)	T3: Ethanol (90%) followed by 5 min 0.1% HgCl_2_ (0.1% Tween-20)

	CFU per seed after seed coat separation			

D	Seed testa homogenization (average 50 mg per seed; in 1 ml FDW) and homogenate CFU monitoring	498 ± 54.07	555 ± 61.7	447 ± 58.5

			Average = 500	

E	Vortexing the perisperm-bearing seed embryos excised from surface disinfected seeds (2 min at top speed) and CFU monitoring	527 ± 78.5	600 ± 40.8	975 ± 120.2

			Average = 700	

F	Monitoring the six sequential washes of excised embryos (with perisperm)	In effect 10^3^ to 2 × 10^3^ CFU was left behind with the seed coat or periderm membrane per seed irrespective of the surface sterilization procedure adopted (average 1,500)		

G	Preparation of embryo homogenate from surface-disinfected seeds and CFU monitoring (after step C)	10^4^ CFU g^–1^ embryo tissue	10^3^ CFU g^–1^ embryo tissue	10^4^ CFU g^–1^ embryo tissue

	Population pattern in “G”	Intermediate to T2 and T3	Mix of sporulating and non-sporulating Gram-positive bacteria	Spore-forming *Bacillus* spp.

*CFU, colony-forming unit; FDW, filter-sterilized autoclaved distilled water.*

The next monitoring step involved drying the above surface-disinfected seeds in a vertical airflow cabinet, removing the hard seed coat aseptically with the help of a nail cutter, and then assessing the testa-homogenate and the embryo parts for any surviving bacteria. The seed testa from surface-sterilized seeds showed substantial bacterial CFU (445–557 per seed), dominantly *Bacillus* spp. The embryos excised from surface-disinfected seeds (with the intact perisperm) bore externally 10^3^ to 10^4^ CFU per embryo as per the six recurrent vortexing washes. The tissue homogenate from perisperm-bearing washed embryos again showed 10^3^ to 10^4^ CFU g^–1^ tissues. Thus, the “supposedly surface-sterilized seeds” harbored a high share of cultivable bacteria inside the seed coat and on the perisperm-bearing embryos. The bacterial population that emerged from the seed testa, embryo washes, or the embryo homogenate varied with the disinfectant employed: slower-growing non-sporulating Gram-positive colony types dominated the NaOCl (4% chlorine) treated set, the HgCl_2_ (0.1%) treated set showed a high population of fast growing *Bacillus* spp., while the mere ethanol-treated seeds showed an intermediate population; the reason for this differential outcome was not understood.

### Embryo Disinfection and Cultivation-Based Assessment of Embryo-Associated Bacteria

**FIGURE 1:**
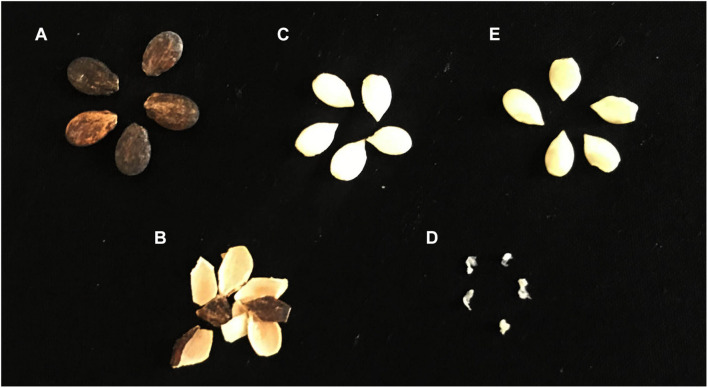
Excision of watermelon seed embryos after seed decoating and surface sterilization, the removal of perisperm tissues, and monitoring the seed embryo homogenate for cultivable bacteria. **(A)** Whole dry seeds, **(B)** removed seed coat, **(C)** seed embryo with perisperm, **(D)** perisperm tissue removed during sodium hypochlorite-mediated surface sterilization, and **(E)** surface-sterilized embryo with the perisperm tissue removed.

The above observations proved that it was essential to go for seed decoating and detailed surface sterilization of excised embryos to ensure the removal of all bacteria external to it. Accordingly, three FDW rinses of excised embryos followed by 90% ethanol (1 min) and 10 min NaOCl (4% chlorine) treatment and six FDW rinses was arrived at as the standard disinfection procedure. This treatment also facilitated the removal of embryo-adhering perisperm (Figure 1). With this procedure, no bacteria were detected in the final wash solutions, in embryo imprints, or in the PCR employing bacterial 16S rRNA universal primers on the two last wash solutions. The homogenate from disinfected embryos did not display any bacterial CFU on NA or TSA during the 1 week of observation except for the grainy raised appearance at the direct-homogenate applied spots re-streaking of which to fresh medium did not elicit any colony growth for another week. Surface-sterilized embryos showed > 90% germination on MS medium indicating that they were viable and healthy. A few bacterial colonies emerged upon the extended incubation of nutrient plates for 2–4 weeks which were not pursued since the emphasis in this study was mainly elucidating the gross embryo association by endophytic bacteria.

### Microscopic Observations on Seed Wash Solutions and Tissue Homogenates

The original seed-vortexed FDW (100 μl per non-disinfected seed) showed a few mobile bacterial cells under live bright field microscopy (×1,000) upon loading 10 μl samples under a 22 × 22 mm cover-glass (Supplementary Movie 1). On the other hand, the seed testa homogenate (50 mg ml^–1^) and the perisperm tissue from surface-sterilized seeds displayed much more/abundant motile bacterial cells (Supplementary Movies 2, 3). Razor-thin tissue sections prepared from re-surface-sterilized embryos (post 1–2 h FDW soak) also exhibited copious motile cocci inside and around the disturbed tissues (Supplementary Movie 4). Tissue homogenate from surface-sterilized embryos (100 mg ml^–1^) appeared as a thick milky suspension with no obvious particle motility but upon dilution (1:10 or 1:100) displayed profuse motile bacteria along with passively moving plastids, mitochondria, and possibly starch grains and other cellular inclusions (Supplementary Movie 5). The embryo homogenate under phase contrast showed phase-bright plastids (≥5 μm) and mitochondria (2–3 μm) along with abundant fine rods and cocci (Supplementary Figure 2), which apparently corresponded to diverse bacteria as elucidated subsequently through 16S rRNA amplicon profiling.

### Microscopy and Fluorescent *in situ* Hybridization on Tissue Sections

**FIGURE 2:**
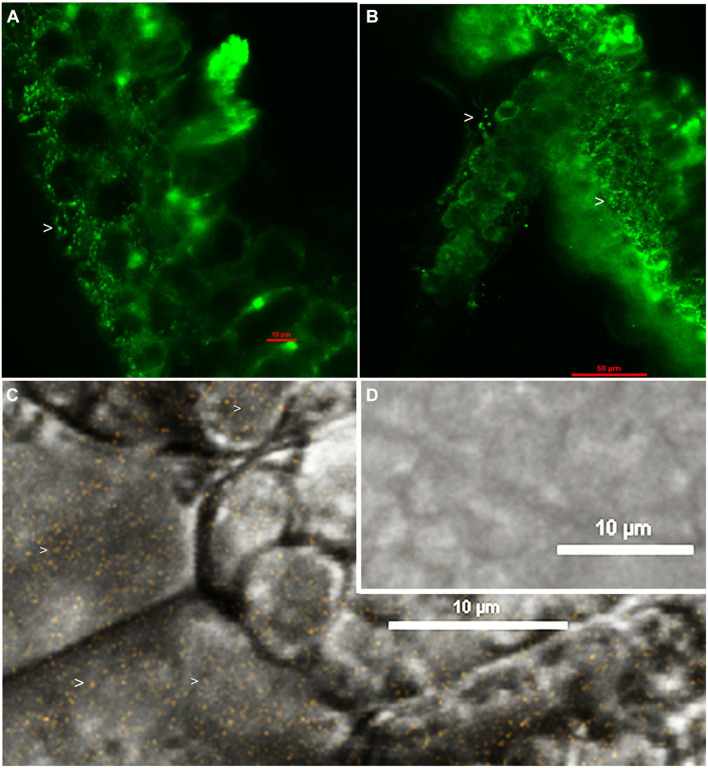
Confocal imaging after SYTO-9 staining, or FISH with Eub338 probe on seed tissue sections of watermelon “Arka Manik”: Perisperm tissue stained with SYTO-9 under 488 nm green channel **(A)**, seed embryo stained with SYTO-9 under 488 nm green channel **(B)**, FISH on seed embryo tissues of watermelon with Cy3 labeled Eub338 displaying abundant fluorescing bacteria under 543.5 nm channel **(C)**, and FISH negative control with Eub338ns **(D)**. Arrow heads show bacterial cells.

Confocal imaging with SYTO-9 staining on perisperm tissue displayed abundant green fluorescing bacteria (Figure 2A). Ultra-thin cryo-sections of seed embryos showed vague SYTO-9 signal (data not shown), while direct embryo sections prepared with a razor blade (after 1–2 h FDW soaking of disinfected embryos) displayed plentiful green-fluorescing bacteria along the cell periphery and in the cytoplasm (Figure 2B). Confocal movie microscopy on SYTO-9 stained seed embryo sections indicated abundant bacteria in the intracellular matrix across different vertical planes (Supplementary Movie 6) in line with bright-field microscopy. FISH employing Eub338 probe displayed abundant bacteria in the intracellular matrix (Figure 2C) with no signal detected in the Eub338ns control (Figure 2D).

### Cultivation Versus 16S Taxonomic Profiling on Watermelon Seeds and Seedlings

#### Watermelon Seed Embryos Versus *in vitro* Seedlings

**FIGURE 3:**
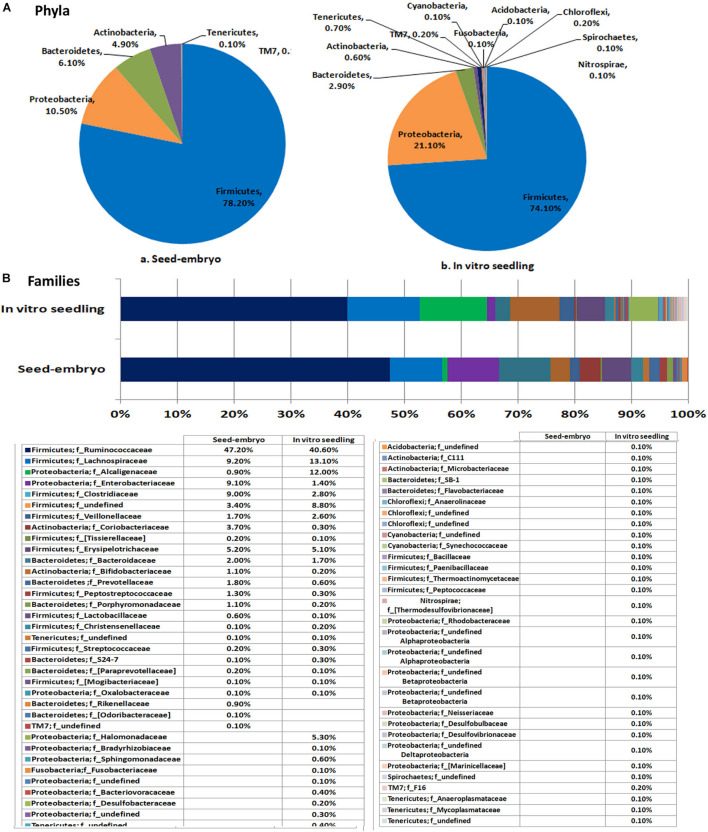
Distribution of phylogenetic groups as per 16S rRNA metagene V3–V4 region taxonomic profiling of DNA derived from the seed embryo versus *in vitro* derived seedlings of watermelon cv. Arka Manik at phylum **(A)** and family **(B)** levels.

This study involved seed embryos and 2-week old seed embryo derived *in vitro* seedlings of watermelon cv. Arka Manik, the latter that were confirmed to be free from cultivable bacteria through their indexing on NA and TSA almost 10 days after culturing the surface-disinfected seed embryos on MS medium. DNA extracted from the milky seed embryo homogenate (sample MG-37) and the index-negative seedlings (MG-41) yielded good amplicon libraries with the 16S rRNA V3–V4 primers (Table 2). After filtering out the reads corresponding to plant and no blast hit sequences as per the QIIME analysis I, QIIME round II analysis on stitched quality reads showed diverse bacteria associated with both seed embryos and *in vitro* grown seedlings. Firmicutes formed the dominant phylum in both samples with similar OTU shares (78.2 and 74.1%, respectively). Seed embryos showed Proteobacteria, Bacteroidetes, and Actinobacteria as the next major phyla, while seedlings displayed an increase in Proteobacterial OTU share, a reduction in Bacteroidetes and Actinobacteria, and the emergence of six additional phyla that were not observed in seed embryos albeit in small shares (Figure 3A). Class level distribution showed 11 constituents in seed embryos and an additional 12 constituents in seedlings again in minor shares (data not shown). Clostridia formed the major class in both seed embryo and seedling samples (72.1 and 68.5%, respectively), with more β-Proteobacteria emerging in seedlings compared with the higher share of γ-Proteobacteria observed with seed embryos. At the family level, the seed embryos showed 26 constituents with Ruminococcaceae as the main constituent followed by Lachnospiraceae, Alcaligenaceae, Enterobacteriaceae, Clostridiaceae, and others, while seedlings displayed notably more diversity (62 constituents) with the dominance of Ruminococcaceae followed by others (Figure 3B). The two samples showed 23 common families with three additional families in seed embryos and 39 extra families in seedlings. At the genus level, undefined Ruminococcacea appeared as the dominant constituent in both samples followed by undefined Enterobacteriaceae, undefined Clostridiaceae, *Faecalibacterium*, and others in seed embryos. Seedlings displayed *Achromobacter*, undefined Clostridiales, *Dorea*, [*Eubacterium*], *Coprococcus*, etc., as the major constituents. Thus, the seedlings displayed a considerably higher diversity compared with seed embryos with 33 common genera, 15 additional genera in seed embryos, and 50 more in seedlings (Supplementary Dataset 1). The results altogether indicated the prevalence of diverse bacteria as embryo-colonizing endophytes and their transmission to new seedlings with taxonomic realignments with respect to the deciphered population. It was significant to note that spore-forming Firmicutes (*Bacillus* and *Paenibacillus* spp.) were not documented in the seed embryos while they formed very minor share in seedlings.

**TABLE 2:** Data statistics for seed embryos (MG37) and 2-week-old in vitro seedlings (MG41) of watermelon “Arka Manik” samples as per QIIME analysis.

Sample name	Seed embryo (MG-37)	*In vitro* seedling (MG-41)
DNA concentration (μg/μl)	33.8	23.0
**QIIME analysis I**		
Number of reads	854,516	722,438
Total data (Mb)	419	341
Mean sequence length	245.18	236.63
Guanine and cytosine (GC)	54%	54%
Stitch reads	774,782	588,705
Mean sequence length of stitch read	457.15	454.9
Number of reads after quality check (QC)	774,501	532,944
Number of OTUs	3,414	6,079
Shannon alpha diversity	4.27	2.77
Number of observed species	4,017	7,095
Taxonomy at phylum level	% OTUs	
Cyanobacteria/chloroplast^*a*^	37.56/37.56	1.30/1.30
Proteobacteria/mitochondria^*b*^	20.59/15.7	1.16/1.09
Firmicutes	36.56	0.27
Actinobacteria	2.28	–
Bacteroidetes	2.87	0.01
No blast hit	0.14	97.25
**QIIME analysis II after removing reads assigned to chloroplast, mitochondria, and unassigned**		
Number of reads removed	441,975	191,434
Total data (Mb)	213	77
Number of stitched reads	362,241	57,701
Number of reads after QC	361,962	1,940
OTUs	3,087	214
Alpha diversity: Shannon index	5.67	5.09
Observed species	3,465	211

*OTU, operational taxonomic unit; QIIME, Quantitative Insights Into Microbial Ecology.*

*^a^Class level.*

*^b^Mitochondria at family level.*

#### Whole Seed Embryos, Embryo Base, and Cotyledons

**FIGURE 4:**
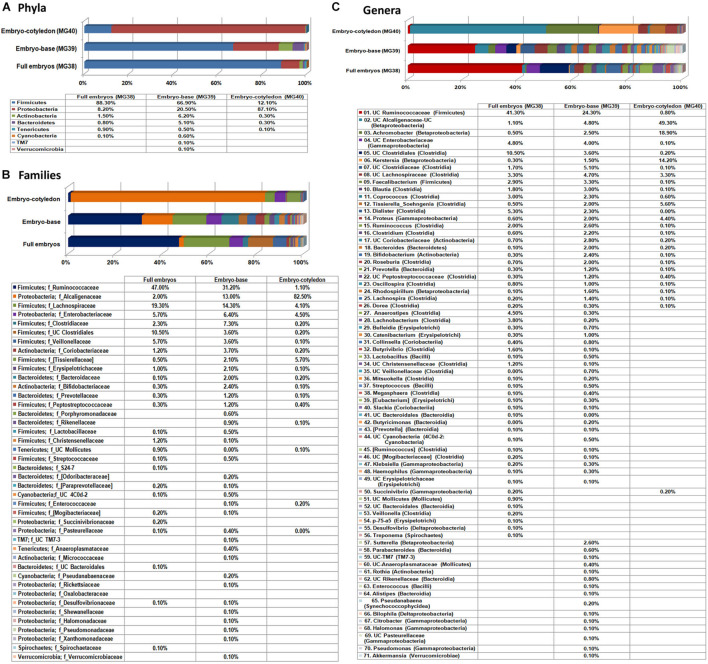
Distribution of phylogenetic groups as per 16S rRNA metagene V3–V4 region taxonomic profiling of DNA derived from the whole excised seed embryos of watermelon “Arka Manik,” seed embryo base and seed embryo cotyledons at phylum **(A)**, class **(B)**, and family **(C)** levels.

In this study, 20 re-disinfected whole seed embryos were employed (MG-38) in comparison with another 20 embryos where the embryo base (radicle-plumule part; MG-39) and cotyledon part (MG-40) were segregated before DNA isolation. After filtering out the reads corresponding to plant and no blast hit sequences as per the QIIME analysis I (Supplementary Table 1), QIIME analysis II on stitched quality reads showed 0.97 million reads for the three samples with 1,825 to 2,444 OTUs. Overall, the embryo base which is to give rise to the root and shoot systems showed more diversity than the cotyledon part, while the whole embryos stood in between (Figure 4). The whole embryos showed an abundance of Firmicutes (88.3%) followed by Proteobacteria as documented earlier for the seed embryo. The embryo base also showed dominant Firmicutes (66.9%) followed by Proteobacteria, Actinobacteria, and Bacteroidetes. The cotyledon part, on the other hand, displayed majorly Proteobacteria (87.1%), while the rest formed mainly Firmicutes (12.1%). The same trend continued at class level with Clostridia constituting the major share for both whole embryo and embryo base which included mainly Ruminococcaceae, and Lachnospiraceae families. The cotyledon part on the other hand largely showed β-Proteobacteria contributed almost exclusively by Alcaligenaceae.

A large share of OTUs under Clostridia and Proteobacteria remained unassigned to any specific genera. The prominent defined genera in whole embryos and the embryo base under Firmicutes included *Faecalibacterium*, *Blautia*, *Coprococcus*, *Soehngenia*, *Dialister*, *Ruminococcus*, *Clostridium*, *Lachnobacterium*, *Roseburia*, and *Anaerostipes*. Other major defined genera included *Achromobacter*, *Kerstersia*, and *Sutterella* under Proteobacteria, *Bacteroides* under Bacteroidetes, and Bifidobacterium under Actinobacteria. The results overall indicated the prevalence of a rich bacterial diversity in watermelon seed embryos with differential population in embryo-base and seed-cotyledon parts and high phylogenic diversity in the radicle-plumule part which is to develop to the new seedlings.

#### Root, Hypocotyl, and Cotyledon Parts of *in vitro* Grown Seedlings

**FIGURE 5:**
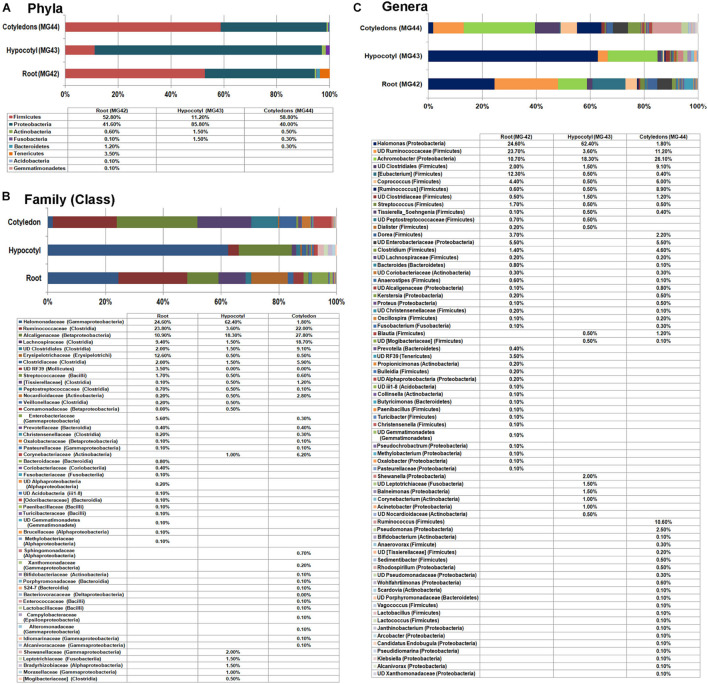
Distribution of phylogenetic groups as per 16S rRNA metagene V3–V4 region taxonomic profiling of DNA derived from the root, hypocotyls, and cotyledon tissues of 2-week old *in vitro* grown seedlings of watermelon “Arka Manik” at phylum **(A)**, family **(B)**, and genus **(C)** levels.

To assess the transmission of bacterial endophytes to the next generation cycle, seedlings derived from the surface-disinfected seed embryos were employed. *In vitro* seedlings growing on Phytagel gelled MS medium after 10 days of embryo culturing were indexed/tested on NA, TSA, and agar-gelled MS medium, and the root, hypocotyl, and cotyledon tissues from individual seedlings were stored singly in 2-ml tubes at −20°C to ensure that only seedlings without any cultivable bacterial association were selected to avoid the over-representation of such bacterial OTUs. Tissues from such 20 seedlings were pooled to three composite samples for the said three parts. QIIME analysis II filtering out the plant sequences showed distinct taxonomic profiles for the three seedling regions (Figure 5). Root tissues displayed the maximum taxonomic diversity (eight phyla) followed by cotyledon (five phyla) and hypocotyl (four phyla). The distribution of major phyla in the root and cotyledon parts appeared similar (Firmicutes 52.0% and 58.8%; Proteobacteria 41.6% and 40.0%). Conversely, hypocotyl showed dominantly Proteobacteria (85.8%) followed by Firmicutes (11.2%). Root tissues also showed a notable share of Tenericutes. At the class level, root tissues showed Clostridia and Erysipelotrichi under Firmicutes, while cotyledon showed a high share of Clostridia. While γ-Proteobacteria formed a major class for the hypocotyls and roots, the cotyledon part showed more of β-Proteobacteria.

At the genus level, both roots and cotyledon parts displayed high diversity (41 and 43 genera, respectively), compared with the hypocotyls (20 genera). Nine genera appeared common to the three samples that constituted 80.1, 64.4, and 88.3% OTUs, respectively, for root, cotyledon, and hypocotyl parts. These included *Halomonas* (γ-Proteobacteria), *Achromobacter* (β-Proteobacteria), [*Eubacterium*], *Coprococcus*, [*Ruminococcus*], *Streptococcus*, *Tissierella_Soehngenia*, undefined Ruminococcaceae, and undefined Clostridia, all belonging to Firmicutes. While the shoot region displayed a high share of *Halomonas* and undefined Ruminococcaceae, the cotyledon part showed a high share of *Achromobacter*. The hypocotyl tissue exhibited high abundance of *Halomonas* and *Achromobacter*. *In vitro* grown seedlings displayed altogether 70 constituents at genus level across the three segments despite the fact that they were protected from all external organisms. This indicated that diverse bacteria are carried by the embryo which in turn transmit to the root and shoot parts of seedlings with differential distribution in various seedling parts. Significantly, *Bacillus* spp. and the related genera of spore formers did not form notable constituents in any part of the seedlings.

#### 16S rRNA Taxonomic V3–V4 Profiling on Different Watermelon Cultivars

**FIGURE 6:**
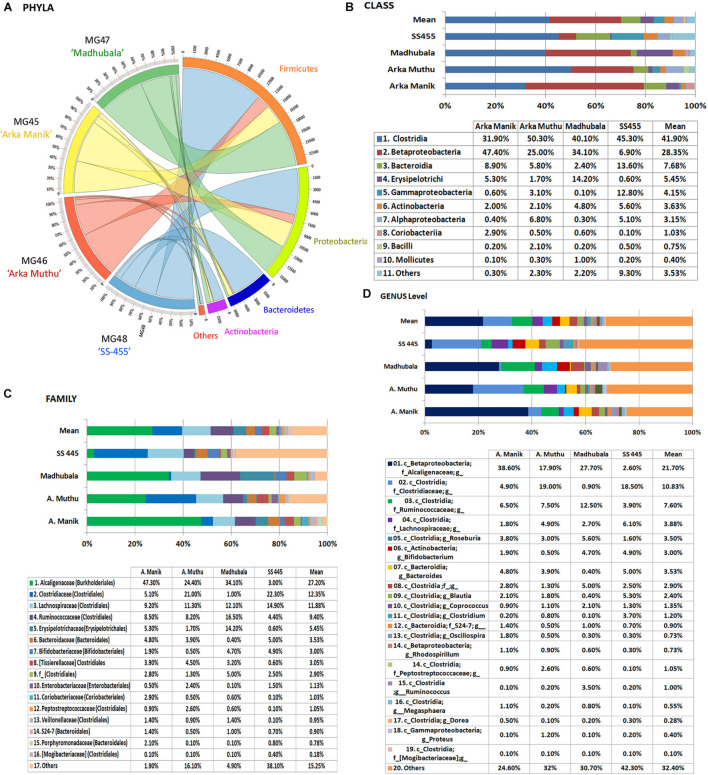
Distribution of phylogenetic groups as per 16S rRNA metagene V3–V4 region taxonomic profiling of DNA derived from the surface-sterilized seed embryos of four watermelon cultivars at phylum **(A)**, class **(B)**, family **(C)**, and genus **(D)** levels.

Embryo homogenates from the four watermelon cultivars (“Arka Manik,” “Arka Muthu,” “Madhubala,” and “SS-455”) did not show any bacterial colony growth on NA/TSA during the 1-week period of observation. This study employed a fresh lot of seeds of “Arka Manik,” while the others were from refrigeration-stored seeds of >6 months. Employing the upper part of the milky embryo homogenates after 1 h standing at 4°C, the DNA yields appeared better than in the previous instance (74.0–110 ng μl^–1^). Illumina sequencing yielded 0.66–0.91 million reads per sample with 0.59–0.84 million stitched reads (Supplementary Table 2). QIIME analysis II excluding the plant sequences gave rise to 3,677–26,157 stitched high quality bacteria-corresponding reads with the OTUs in the range of 395 (“Arka Muthu”) and 1,574 (“SS-455”). Firmicutes formed the main phylum in three cultivars similar to the observations documented earlier with “Arka Manik.” Conversely, cv. Arka Manik in this trial showed Proteobacteria as the dominant phylum which possibly arose from the use of a fresh seed lot as documented with other fresh seed samples also (unpublished data). The four phyla including Firmicutes, Proteobacteria, Actinobacteria, and Bacteroidetes constituted a core microbiome of 96.9–99.6% OTUs in the four cultivars (Figure 6A). Minor shares of 17 other phyla were seen in one or more cultivars. At the class level, the four cultivars showed OTU distribution under 40 constituents (Supplementary Dataset 2) with 10 common classes constituting ≥ 95% OTUs (Figure 6B).

The OTUs were distributed under 23, 58, 26, and 72 families, respectively, for the above four cultivars with 16 core families constituting 98.1% (“Arka Manik”), 83.9% (“Arka Muthu”), 95.1% (“Madhubala”), and 61.9% (“SS-455”) OTUs in them (Figure 6C). Alcaligenaceae, Clostridiaceae, Ruminococcaceae, and Lachnospiraceae were seen across the four cultivars as major families, while Xanthomonadaceae, Sphingomonadaceae, and a few other families accounted for the difference in “SS-455.” Genus level distribution indicated a huge bacterial diversity spanning across 159 units with 19 core genera accounting for 75.4, 68, 69.3, and 57.7% OTUs, respectively, in the four cultivars (Figure 6D). The core genera included unclassified Alcaligenaceae, *Rhodospirillum* (β-Proteobacteria), *Proteus* (γ-Proteobacteria), and several unclassified groups. The maximum amount of diversity was observed under class Clostridia with 31 genera and 14 core genera. Only negligible amounts of spore-forming genera (0.1 *Bacillus* spp. in “Arka Manik,” and 0.5% in “Arka Muthu,” 0.2% *Paenibacillus* in “Arka Muthu,” and 0.1% *Lactobacillus* in “SS-445”) were documented in the embryo tissues.

## Discussion

Seeds in dicotyledonous plants grow to maturity well protected inside fruit tissues. Under natural conditions, the fruit degenerates releasing the mature seeds to the environment. Under organized cultivation, the seeds are processed and utilized soon after, or maintained in storage. Thus, the seeds could bear the organisms inherited from the mother plant, if any, besides those acquired from environment (Nelson, 2018; Rodríguez et al., 2018). As the seed germinates, microorganisms from three sources get to associate with it: (i) external microbiota from the seed spermosphere, (ii), seed internal microbiome from the testa and perisperm, and (iii), the true embryo colonizers (Nelson, 2018). While the former two categories of microorganisms could converge as endophytes in new seedlings, the microbiome transmitted from the parent through the ovum plus that contributed by pollen (Maniranjan et al., 2017) which reaches the embryo alone could qualify as vertically transmitted organisms that are certain to be passed on to the seedlings (Abdelfattah et al., 2021). The present investigations reveal a vast diversity of bacterial phyla and genera prevailing inside watermelon seed embryos with clear vertical transmission prospects and assured colonization of new seedling (Abdelfattah et al., 2021). The quantum of such deep-seated cultivation-recalcitrant microbes elucidated in this study is much higher than the generally documented diversity (Hameed et al., 2015; Robinson et al., 2016; Adam et al., 2018) in line with the recent report on oak seed embryos which also demonstrate the differential transmittance of microbiome to the roots and phyllosphere of new seedlings (Abdelfattah et al., 2021). These intimately plant-associated microbes will hold considerable significance in deciding the colonization competence, survival, and functioning of the native rhizospheric microbiome as well as any laterally applied inocula.

The major limitation while studying the vertical transmission of endophytic bacteria is the intricacy in dissecting out the embryos distinctly and growing the seedlings away from environmental microorganisms. Several past studies have addressed the seed microbiome and seed endophytes most of which employed mere surface-sterilized seeds with no discrimination between the embryo and the external tissues (Khalaf and Raizada, 2016; Bergna et al., 2018). Most plant species that have been the subjects of seed microbiome studies showed the firm attachment of embryo to the seed coat such as in *Arabidopsis*, legumes, maize, cotton, brassicas, tobacco, etc. Microscopic observations in this study have shown a substantial bacterial load in watermelon seed testa and perisperm tissues even after extensive surface sterilization steps. Monitoring the wash solutions and seed imprints in this study ensured effective surface sterilization. Past cultivation-based studies on embryos excised from surface-sterilized seeds often showed a significant amount of spore-forming bacteria (Truyens et al., 2015; Nelson, 2018). The re-disinfected embryos in this study displayed no readily cultivable bacteria on two common bacteriological media for a reasonable period of 1 week which indicated that the organisms survived as CREBs.

Considering the literature available on seed microbiome, very few studies have considered the seed embryo tissues distinct from other seed parts. Analyzing the seed endosphere of four rice cultivars after surface sterilization of de-hulled seeds (without discriminating embryo and endosperm tissues) through cultivation versus cultivation-independent PCR-denaturing gradient gel electrophoresis (DGGE), Hameed et al. (2015) observed a total of 20 distinct PCR-DGGE bands most of which corresponded to γ-Proteobacteria (50%) followed by *Bacilli* (25%) and β-Proteobacteria (10%). In wheat, where the embryo and endosperm were separated out, Robinson et al. (2016) found the bacterial association only with the endosperm and not in the embryo. Cultivation-independent analysis of bacterial biome distinctly in the endosperm and germ tissues of wheat seeds showed several beneficial bacterial genera in the endosperm tissues and a lesser diversity in the germ tissues (Kuźniar et al., 2020a). Further exploration on seeds of different wheat cultivars demonstrated the embryo transmission of about 20 genera with 10 genera shared with the 35 genera documented for the endosperm (Kuźniar et al., 2020b) and the differential distribution of various genera in different organs (root, coleoptile, and leaf) of field plants (Kuźniar et al., 2020a, b). The easy decoating of watermelon seeds unlike wheat seeds which needed extended water-soaking for the embryo dissection (with possible modifications in microbial profiles), excluding the maternal perisperm membrane, allowed a clear conclusion on vertical transmission of diverse endophytic bacteria. The study employing oak acorn excised embryos and seedlings raised under special conditions showed a high microbial diversity and spatial partitioning of fungal and bacterial communities within both seed and seedling indicating vertical inheritance, niche differentiation, and divergent transmission routes for the establishment of root and phyllosphere communities (Abdelfattah et al., 2021). The observations also amend the present understanding depicting soil/environment as the main source of endophytic microorganisms.

This study was directed at bringing out the maximum amount of taxonomic diversity including the minor OTUs to understand the true seed transmission of endophytes rather than the common practice of elucidating the functional aspects of major associates. Firmicutes formed the dominant phylum inside the dry seed embryos followed by Proteobacteria, Actinobacteria, and Bacteroidetes along with minor shares of a number of other phyla including several candidate phyla that lack the cultured relatives. On the other hand, embryos from seeds which were not in long-term storage showed a predominance of Proteobacteria. This appeared the case for freshly harvested seeds which did not go through the desiccation (Thomas, unpublished data). This indicated that during seed drying, the share of Proteobacteria goes down leading to a larger share of Firmicutes. Class Clostridia formed the major constituent under Firmicutes which was understandable considering the anaerobic conditions prevailing inside dry seeds. *In vitro* grown seedlings showed a different phylogenetic profile from seed embryos indicating a taxonomic realignment with seed germination and seedling growth and a variable distribution within different seedling parts. The variable taxonomic profiles for the embryos of different watermelon cultivars and for different seedling parts suggested the prevalence of a much higher diversity of bacterial biome which is perhaps dynamic and varying as per the growth phase or the prevailing conditions such as fresh or older seeds, dry or soaked seeds, etc.

Spore-forming bacterial genera are commonly documented as seed-associated organisms and often considered as seed endophytes in cucurbits (Khalaf and Raizada, 2016) and other crops (Truyens et al., 2015; Nelson, 2018; Kuźniar et al., 2020b). Considering the low moisture content and the high osmotic potential on and inside dry seeds, endospore formation is considered as an important feature for seed colonizers (Truyens et al., 2015; Cope-Selby et al., 2017; Shahzad et al., 2018). Cultivation-based studies on seed testa and perisperm tissues of watermelon in this investigation indicated a huge share of *Bacillus*/other spore formers (often documented with the surface-sterilized seeds) which clearly emanated from seed external tissues. Deep-sequencing studies on seed embryos showed only very negligible share of spore-forming genera (≤1%) across different watermelon cultivars similar to the 16S rRNA V4 profiling study on surface-washed seeds of different pumpkin genotypes where *Bacillus* sp. constituted a very minor (∼1%) share (Adam et al., 2018). Molecular studies on tomato seeds also showed *Bacillus* spp. as a minor constituent, while *Bacillus* and *Paenibacillus* spp. formed major cultivable bacteria (Bergna et al., 2018; Thomas and Shaik, 2020). Deep-sequencing-based studies on surface-sterilized tomato seeds further showed a huge bacterial taxonomic diversity with very identical OTU profiles for two cultivars, while spore-forming Firmicutes formed only a very low share (Thomas and Shaik, 2020). Thus, the observations with the excised cucurbit embryos suggested that spore-forming genera do not form major vertically transmissible organisms.

It was also worth noting the survival of diverse genera of Gram-negative Proteobacteria and Bacteroidetes and non-spore-forming Actinobacteria inside dry embryos under high desiccation. Several bacteria are known to enter viable but non-cultivable (VBNC) state with them turning cultivable upon the return of suitable conditions (Barer et al., 1993; Ramamurthy et al., 2014). The term “cultivation recalcitrant endophytic bacteria” (Thomas and Shaik, 2020) best describes such endophytic bacteria, some of which could be brought to cultivation with specialized media or in the presence of host tissue constituents (Thomas, 2011; Thomas and Franco, 2021). It is common to observe the activation of normally uncultivable bacteria to cultivation during micropropagation or other tissue culture applications as documented with banana (Thomas et al., 2008), watermelon (Thomas, 2011), etc. Recent observations with tomato seeds cultured *in vitro* where the seed coat was removed post-germination showed the gradual activation of different bacteria that constituted mainly Gram-negative Proteobacteria and Bacteroidetes and some Gram-positive non-spore-forming Actinobacteria (Shaik and Thomas, 2019). Cultivation-independent 16S V3–V4 taxonomic profiling on surface-sterilized seeds of tomato had shown a huge diversity of CREB which included mainly Proteobacteria followed by Firmicutes, Actinobacteria, and Bacteroidetes. *Bacillus* and other spore formers appeared as predominant seed external associates with a very minor share of OTUs recorded in molecular analysis (Thomas and Shaik, 2020).

How the organisms reach the inside of the seeds is a vital aspect. Endophytes are known to gain entry inside plants mainly through roots and through natural openings and wounds from phyllosphere and other aerial plant parts from where they colonize the vascular stream and reach various plant organs (Compant et al., 2011; Hardoim et al., 2015). Bacterial endophytes are considered to be transmitted inside seeds from vegetative parts through various routes such as vascular connections traversing the micropyle, colonizing the shoot meristems that transforms to floral parts, through horizontal movement inside the fruits, or through pollen (Truyens et al., 2015; Berg and Raaijmakers, 2018; Nelson, 2018). Obligate and strict vertical transfer of bacteria is considered unlikely in plants (Frank et al., 2017). In melon, some amount of bacteria is considered to enter the fruit from vegetative parts and from there to seeds in the early stages of seed development wherein the thin envelope enclosing the embryo is considered to act as a barrier for bacteria in the later stages of seed maturation (Khalaf and Raizada, 2016).

Transmission of endophytic bacteria through pollen has been established in different plant species (Maniranjan et al., 2017), which allows their direct passage to the embryo. In our assessment, vertical transmission through gametes or seeds essentially needs the organisms to be able to colonize the intracellular niche of tissues that contribute to pollen or ovum development. Endophytic bacteria are considered primarily colonizers in the intercellular region (Hardoim et al., 2015; Alibrandi et al., 2018). Microscopic observations on banana and papaya have indicated abundant cytoplasmic colonization by endophytic bacteria with their terming as “Cytobacts” (Thomas and Sekhar, 2014; Thomas et al., 2019) in which case the intracellular bacteria could move to the gametophytes through mitosis and meiosis. Intracellular bacteria have also been documented in the meristem of pine (Pirttilä et al., 2000) that could move to floral tissues and reproductive units (Frank et al., 2017) and also in axenically grown pineapple and orchids (Esposito-Polesi et al., 2017). More recent studies implying cell cultures of grapevine and other plant species have shown abundant and diverse Cytobacts across plant species with their origin ascribable to the field source tissues (Thomas and Franco, 2021) with clear indication of vertical transmission across generations (Thomas et al., unpublished data). It is also possible that bacteria from the stigma get incorporated to the embryo at fertilization (Mitter et al., 2017).

The seed embryo microbiomes are likely participating in various plant processes including growth promotion, host defense, and metabolic pathways. It is understood that functional elucidation of seed endophytes is not practically easy considering that the associated organisms are diverse and uncultivable, their dynamic and variable nature, inability to focus on single organisms at the exclusion of others, and variable population structure depending on the organ and the developmental stage of seedlings (Shaik and Thomas, 2019). The functional elucidations would warrant concerted efforts by different research groups. The recent report that the individual seeds of bean and radish were associated with a dominant bacterial taxon which in turn was highly variable between plants and within seeds of the same plant is worth noting (Chesneau et al., 2021). The embryo-associated bacteria have the advantage of being able to establish and spread to different parts of seedlings at germination before the externally associated microorganisms make their way inside seedlings. Some endophytic bacteria can also get out of the plant and colonize the rhizosphere (Johnston-Monje and Raizada, 2011), which also applies to embryo-derived endophytes (Abdelfattah et al., 2021). The interactive effects between the internal versus external organisms at seed germination, with seedling growth and the selective acquisition of organisms from the spermosphere or rhizosphere, are worthy of in-depth investigations.

It is now certain that the embryos are coming packaged with a series of endophytic bacteria which certainly have the edge over external organisms (Khalaf and Raizada, 2016). Seed-associated microbes can improve seed germination, promote seedling health, enhance plant growth, and mitigate stress (Shahzad et al., 2018). As the concept of holobiome highlights the inseparable significance of the microbiome in developmental and other physiological behavior of the individual (Kim and Lee, 2020), which has been well proven by the studies on the gut microbiome, the phytobiome could be a panacea for solving the emerging problems in crop production. Keeping in mind that plant microbiome is proposed as a platform for realizing the next green revolution (Rodriguez and Durán, 2020), deciphering the native/seed transmitted endophytes could be of greater significance. Thus, this study would be pivotal in widening our understanding of the structure and transmission of plant microbiome and gathering insights for their roles in plant growth and health promotion.

In summary, the deep sequencing and microscopy-based investigations on watermelon seed embryos revealed abundant and enormously diverse bacteria colonizing the seed embryo tissues and transmitted to the seedlings, and in all probability vertically to the next cycle. The extent of bacterial diversity documented within the seed embryos clearly excluding the seed coat parts was unprecedented unlike as documented in the earlier published reports. The results here indicate that the seeds/embryos come packaged with their microbiome which spread to different parts of the developing seedling/plant unlike the earlier understanding that the plants mostly acquired the desirable endophytic microorganisms from the soil/rhizosphere. This embryo colonization by bacteria might be facilitated by the intracellularly associated diverse “Cytobacts.” It calls for more in-depth investigations to understand the entry routes of endophytes inside seed embryos and how the organisms get activated/multiply and distribute themselves to the root/shoot tissues of the new plant besides their functional roles. The well-protected seed embryos inside the seed coat in watermelon and other cucurbits with their prominent embryos and the feasibility of removing the testa form ideal candidates to study vertically transmitted bacterial endophytes.

## Data Availability Statement

The datasets presented in this study can be found in online repositories. The names of the repository/repositories and accession number(s) can be found in the article/Supplementary Material.

## Author Contributions

PT: conceiving the idea, conduct of the experiments, data analysis and interpretation, and manuscript preparation. PS: undertaking confocal microscopy and FISH (fluorescent *in situ* hybridization). Both authors contributed to the article and approved the submitted version.

## Conflict of Interest

PT has been employed at the company Thomas Biotech & Cytobacts Centre for Biosciences (OPC) Pvt. Ltd., Bengaluru, India; and is currently acting as the CEO & Director of this start-up. The remaining author declares that the research was conducted in the absence of any commercial or financial relationships that could be construed as a potential conflict of interest.

## Publisher’s Note

All claims expressed in this article are solely those of the authors and do not necessarily represent those of their affiliated organizations, or those of the publisher, the editors and the reviewers. Any product that may be evaluated in this article, or claim that may be made by its manufacturer, is not guaranteed or endorsed by the publisher.

## Footnotes

1
www.xcelrislabs.com

## Supplementary Material

Supplementary Figure 1 Assessing seed external bacterial load and the efficacy of three surface sterilization treatments (ethanol treatment; ethanol + NaOCl, ethanol + HgCl_2_) through bacterial monitoring on nutrient agar (NA) after different treatment steps with *Bacillus/other* spore-forming bacteria detected after different steps. **(A)** SP-SDS of seed first wash solution at the rate of 100 μl per seed, with the lowest dilution on the top left side and highest dilutions on the right lower side **(B)** monitoring of six washes through spotting 10 μl solutions, **(C)**, bacterial monitoring after ethanol step and five washes, **(D)** SP-SDS of seed testa homogenate, **(E)** SP-SDS of excised embryos dispersed in FDW, **(F)** monitoring the six sequential washes of excised embryos with perisperm, and **(G)** SP-SDS of embryo homogenate.

Supplementary Figure 2 Seed embryo homogenate from surface-sterilized seed embryos of watermelon under phase contrast (100× objective) displaying starch grain aggregates (s), large phase bright plastids of > 5 μm size (p) and mitochondria of 2–3 μm size (m) along with fine bacterial cells of ≤ 1 μm (b) in the background.

Supplementary Table 1 Data Statistics for watermelon Seed Embryos-Bulk (MG38), Embryo base (MG39) and Embryo cotyledons (MG40) from 40 seeds each as per QIIME analysis I and II.

Supplementary Table 2 DNA yields and 16S rRNA V3-V4 region based Illumina sequencing and QIIME- bioinformatics analysis on four watermelon cultivars.

Supplementary Dataset 1 OTU abundance at genera level in watermelon seed embryo (MG37) and *in vitro* derived seedlings (MG41).

Supplementary Dataset 2 OTU abundance at class level in different cultivars of watermelon.

Supplementary Movie 1 Bright field microscopy (1000×) on the original seed-vortexed FDW (100 μl per non-disinfected seed) showing few mobile bacterial cells (10 μl sample loaded under a 22 × 22 mm cover-glass).

Supplementary Movie 2 Bright field microscopy (1000×) on the seed-testa homogenate (50 mg ml^–1^) from surface sterilized seeds displaying abundant motile bacterial cells (10 μl sample loaded under a 22 × 22 mm cover-glass).

Supplementary Movie 3 Bright field microscopy (1000×) on perisperm tissue from surface sterilized seeds (one per 50 μl) displaying abundant motile bacterial cells (10 μl sample loaded under a 22 × 22 mm cover-glass).

Supplementary Movie 4 Razor thin tissue sections prepared from re-surface sterilized embryos (post 1–2 h FDW-soak) exhibiting copious motile bacterial cocci inside and around the disturbed tissues.

Supplementary Movie 5 Milky tissue homogenate from surface sterilized embryos of ‘Arka Manik’ watermelon (10 mg ml^–1^) displaying profuse motile bacteria along with passively moving plastids and mitochondria under bright field microscopy (1000×).

Supplementary Movie 6 Confocal Movie-microscopy on SYTO-9 stained seed-embryo sections showing abundant bacteria in the intracellular matrix across different vertical planes.

## Abbreviations

CREB: cultivation recalcitrant endophytic bacteria
FDW: filter-sterilized autoclaved distilled water
MS medium: Murashige and Skoog (1962) medium
NA: nutrient agar
OTU: operational taxonomic unit
SATS: spotting-and-tilt-spreading
SP-SDS: single plate-serial dilution spotting
STH: seed tissue homogenate
TSA: trypticase soy agar
VBNC: viable but non-cultivable.
