# Supplementary figures and images for: Vertical Transmission of Diverse Cultivation-Recalcitrant Endophytic Bacteria Elucidated Using Watermelon Seed Embryos
Source: Front Microbiol. 2021 Nov 15;12:635810. doi: 10.3389/fmicb.2021.635810 (PMC8634838; doi:10.3389/fmicb.2021.635810)

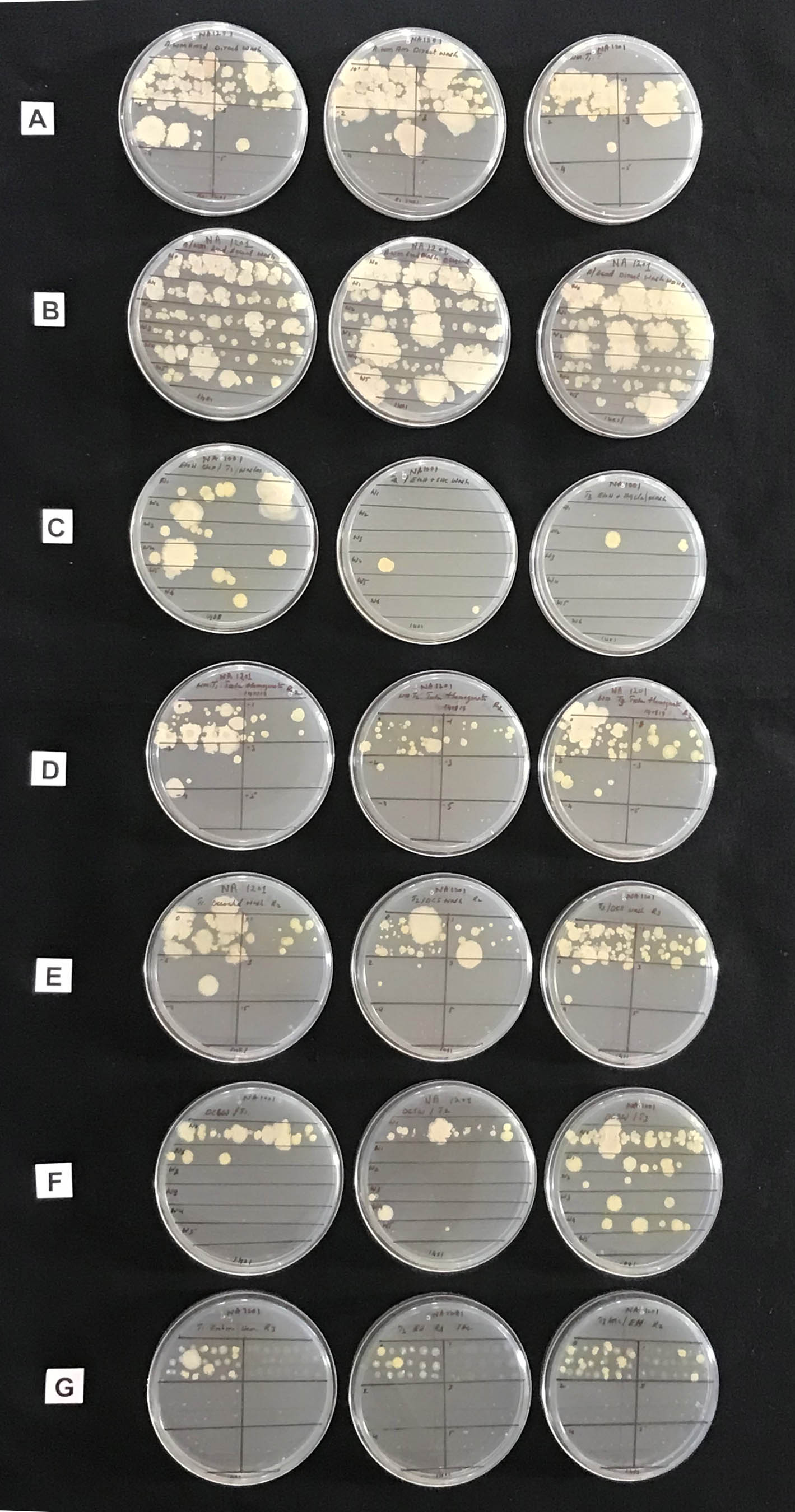

Supplement: Supplementary Figure 1 — Assessing seed external bacterial load and the efficacy of three surface sterilization treatments (ethanol treatment; ethanol + NaOCl, ethanol + HgCl2) through bacterial monitoring on nutrient agar (NA) after different treatment steps with Bacillus/other spore-forming bacteria detected after different steps. (A) SP-SDS of seed first wash solution at the rate of 100 μl per seed, with the lowest dilution on the top left side and highest dilutions on the right lower side (B) monitoring of six washes through spotting 10 μl solutions, (C), bacterial monitoring after ethanol step and five washes, (D) SP-SDS of seed testa homogenate, (E) SP-SDS of excised embryos dispersed in FDW, (F) monitoring the six sequential washes of excised embryos with perisperm, and (G) SP-SDS of embryo homogenate. [file Image_1.JPEG]

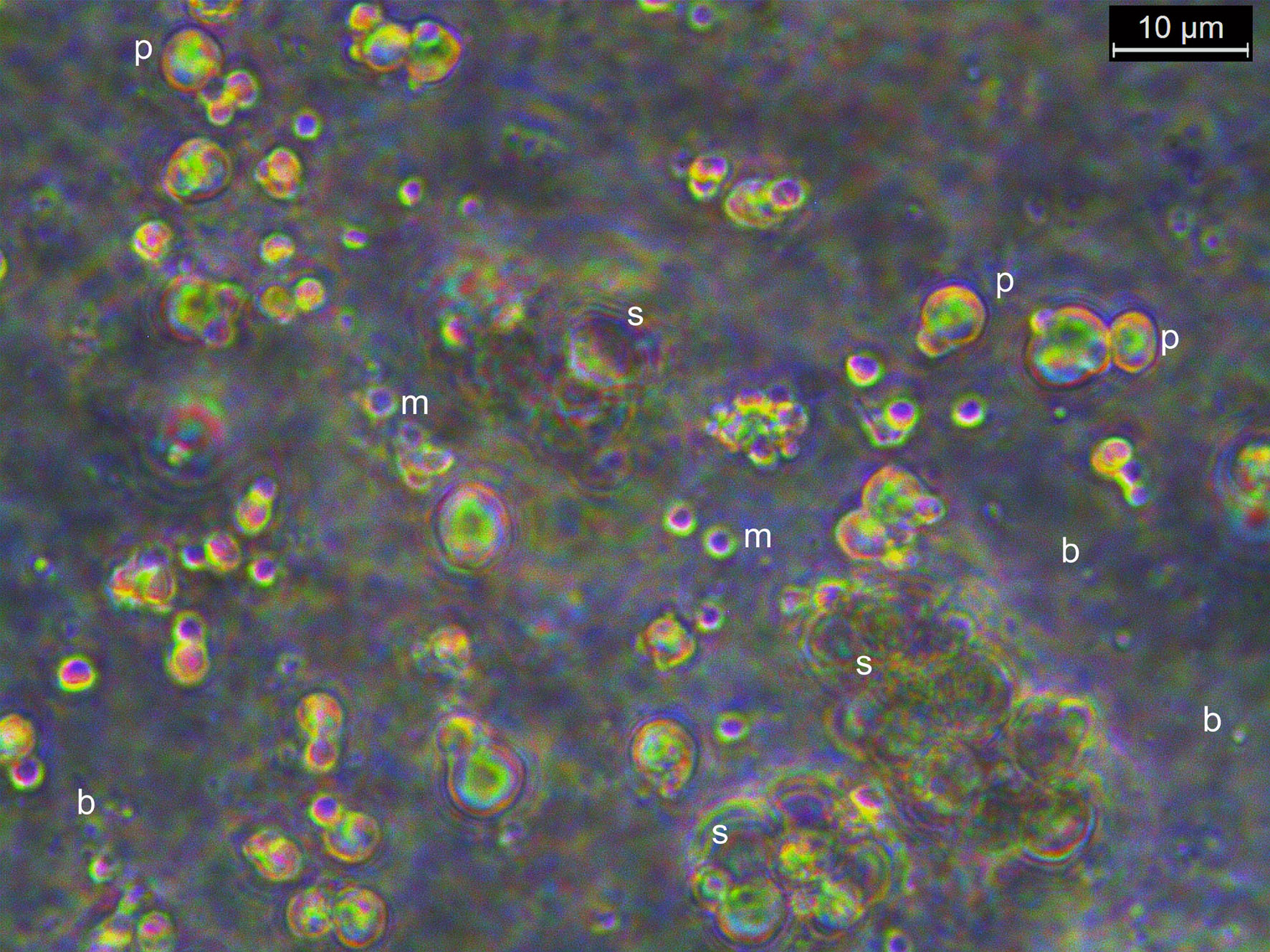

Supplement: Supplementary Figure 2 — Seed embryo homogenate from surface-sterilized seed embryos of watermelon under phase contrast (100× objective) displaying starch grain aggregates (s), large phase bright plastids of > 5 μm size (p) and mitochondria of 2–3 μm size (m) along with fine bacterial cells of ≤ 1 μm (b) in the background. [file Image_2.JPEG]
