# Supplementary material for: Vertical Transmission of Diverse Cultivation-Recalcitrant Endophytic Bacteria Elucidated Using Watermelon Seed Embryos
Source: Front Microbiol. 2021 Nov 15;12:635810. doi: 10.3389/fmicb.2021.635810 (PMC8634838; doi:10.3389/fmicb.2021.635810)
Supplement: Supplementary Table 1 — Data Statistics for watermelon Seed Embryos-Bulk (MG38), Embryo base (MG39) and Embryo cotyledons (MG40) from 40 seeds each as per QIIME analysis I and II. [file Table_1.docx]

**SUPPLEMENTAL TABLES**

**Table S1.** Data Statistics for watermelon Seed Embryos-Bulk (MG38), Embryo base (MG39) and Embryo cotyledons (MG40) from 40 seeds each as per QIIME analysis-I and II.

| Sample Name | Seed embryos-Bulk (MG38) | Seed embryo base (MG39) | Seed embryo cotyledons (MG40) |
| --- | --- | --- | --- |
| DNA concentration (μg/ μl) | 32.2 | 43.4 | 45.2 |
| **QIIME analysis-I** |  |  |  |
| No. of reads | 794,234 | 853,194 | 731,666 |
| Total Data (Mb) | 387 Mb | 418 Mb | 358 Mb |
| Mean Seq length | 244.16 | 245.06 | 245.07 |
| GC (%) | 54% | 54% | 52% |
| Stitch reads | 706,239 | 777,415 | 637,124 |
| Mean Seq length of Stitch Read | 455.28 | 455.11 | 471.41 |
| No. of reads after QC | 706,023 | 777,157 | 636,636 |
| **QIIME Analysis-I** |  |  |  |
| No. of OTUs | 2,735 | 2,389 | 3,041 |
| **Shannon alpha diversity** | 3.09 | 1.28 | 3.25 |
| **# Observed species** | 3115.0 | 2767.0 | 3970.0 |
| **Taxonomy at PHYLUM level (% OTUs)** |  |  |  |
| Cyanobacteria/ Chloroplast^†^ | 53.20/ 53.20 | 79.40/ 79.40 | 16.20/ 16.20 |
| Proteobacteria / Mitochondria | 25.80/ 24.10 | 17.60/ 16.80 | 73.10/ 0.60 |
| Firmicutes | 18.70 | 2.60 | 10.10 |
| Actinobacteria | 0.30 | 0.20 | 0.20 |
| Bacteroidetes | 0.20 | 0.20 | 0.30 |
| **QIIME analysis-II** |  |  |  |
| Reads removed (assigned to chloroplast, mitochondria & unassigned) | 5,45,488 (68.68%) | 7,47,104 (87.56%) | 1,06,523 (14.56%) |
| Total Data (Mb) | 116 | 46 | 305 |
| No. of reads | 248,746 | 106,090 | 625,143 |
| No. of stitched reads | 160,751 | 30,311 | 530,601 |
| No. of reads after QC | 160,535 | 30,050 | 529,640 |
| OTUs | 2,444 | 2,078 | 1,825 |
| Alpha diversity: Shannon index | 6.25 | 7.62 | 2.64 |
| Observed species | 2603 | 2370 | 2067 |

^†^Class level; ^‡^mitochondria at family level
