# Supplementary material for: Vertical Transmission of Diverse Cultivation-Recalcitrant Endophytic Bacteria Elucidated Using Watermelon Seed Embryos
Source: Front Microbiol. 2021 Nov 15;12:635810. doi: 10.3389/fmicb.2021.635810 (PMC8634838; doi:10.3389/fmicb.2021.635810)
Supplement: Supplementary Table 2 — DNA yields and 16S rRNA V3-V4 region based Illumina sequencing and QIIME- bioinformatics analysis on four watermelon cultivars. [file Table_2.docx]

**SUPPLEMENTAL TABLE**

**Table S2**. DNA yields and 16S rRNA V3-V4 region based Illumina sequencing and QIIME- bioinformatics analysis on four watermelon cultivars.

| Particulars | Arka Manik (MG45) | Arka Muthu (MG46) | Madhubala (MG47) | ‘SS455’ (MG48) |
| --- | --- | --- | --- | --- |
| DNA yield (ng/μl) | 110 | 102 | 90.8 | 74.0 |
| **QIIME analysis-I** |  |  |  |  |
| No. of reads | 823,275 | 659,063 | 857,226 | 911,479 |
| Total Data (MB) | 402 | 321 | 419 | 447 |
| Mean seq length | 244.65 | 243.78 | 244.59 | 245.50 |
| Stitch reads | 746,412 | 587,699 | 776,329 | 836,080 |
| Mean seq length of Stitch Reads | 455.13 | 454.93 | 455.14 | 455.34 |
| No. of reads after QC | 746,131 | 576,924 | 776,058 | 835,732 |
| No. of OTUs | *6,266* | *7,270* | *6,517* | *3,747* |
| Common OTUs |  |  | *26,955* |  |
| **Phylum level taxonomy** | OTUs (%) | | | |
| No blast hit | 92.20 | 96.10 | 96.90 | 85.0 |
| Cyanobacteria/ chloroplast | 3.30 | 2.50 | 0.60 | 0.10 |
| Proteobacteria/ Ricketsiales/ mitochondria^‡^ | 4.0/3.5 | 1.0/0.8 | 1.5/1.0 | 12.6/ 11.8 |
| Firmicutes | 0.40 | 0.30 | 0.90 | 1.50 |
| Actinobacteria |  |  |  |  |
| Bacteroidetes | 0.10 | 0 | 0 | 0.60 |
| **QIIME analysis-II** |  |  |  |  |
| Reads assigned to chloroplast, mitochondria and no blast hits and removed | 738,574 | 573,247 | 763,733 | 809,575 |
| PE reads selected | 84,701 | 85,816 | 93,493 | 101,904 |
| Total Data (MB) | 34 | 36 | 38 | 44 |
| Stitched reads | 7,838 | 14,452 | 12,596 | 26,505 |
| Quality reads | 7,556 | 3,677 | 12,323 | 26,157 |
| No. of OTUs | 471 | 395 | 519 | 1,574 |
| Common OTUs |  |  | 2,769 |  |
| Shannon **alpha diversity** | 4.78 | 6.07 | 5.01 | 6.46 |
| # Observed species | 523 | 430 | 559 | 1,589 |
| **Beta Diversity** |  |  |  |  |
| MG45 | 0 | 2402.84 | 1796.86 | 5766.77 |
| MG46 |  | 0 | 3382.01 | 4971.50 |
| MG47 |  |  | 0 | 6155.26 |
| MG48 |  |  |  | 0 |
